# Reply to ‘Inconclusive evidence for rapid adaptive evolution’

**DOI:** 10.1038/s41467-018-05120-9

**Published:** 2018-07-10

**Authors:** Camilla Lo Cascio Sætre, Charles Coleiro, Martin Austad, Mark Gauci, Glenn-Peter Sætre, Kjetil Lysne Voje, Fabrice Eroukhmanoff

**Affiliations:** 1 Department of Biology, Centre for Ecological and Evolutionary Synthesis, University of Oslo, P. O. Box 1066 Blindern, 0316 Oslo, Norway; 2 BirdLife Malta, Xemxija Waterfront Apartments, Flat ½, Triq Is-Simar, Xemxija, SPB 9025 Malta

In our study^1^, we showed that a newly founded population of reed warblers in Malta had undergone a decrease in body mass through 19 years, following a trajectory consistent with a population ascending an adaptive peak, an Ornstein–Uhlenbeck process (OU)^2^. Neto et al.^3^ claim that our result is an artifact of including migrants in the dataset, which inflated the average body mass in the initial years. Controlling for possible seasonal effects is important, which we thank Neto et al.^3^ for pointing out. We now control for season in three different ways and the OU-model always fits better than the neutral model, further strengthening our original conclusion of adaptive evolution.

In Malta, the autumn migration mainly takes place in September, and spring migrants arrive in April or early May^4^. We think limiting our data to mid-June to mid-July, as Neto et al.^3^ suggest, is unreasonably restricted for capturing local birds. We chose to include the entire breeding season (May–August)^4^, as it has been described in several other studies^5–7^. We cannot exclude the possibility that there are some migrants in our dataset, but we investigate possible biases in our sampling design. The ratio of birds captured in the center of the breeding season (June and July) to birds captured in May and August, where the possibility of migrants is greater, shows no apparent trend throughout the years (Supplementary Figure 1). However, body mass is significantly correlated with capture date (linear regression: *R*^2^ = 0.23, *P* *<* 2e−16; Supplementary Figure 2). Thus, we agree there is a need to correct for capture date in our models.

We controlled for capture date in three different ways, and in all three cases, the OU-model outcompeted the Random walk (neutral) model (see Supplementary Table 1, Fig. 1). In fact, some of the new results show an even larger difference in relative model fit than in our initial study.

**Fig. 1:**
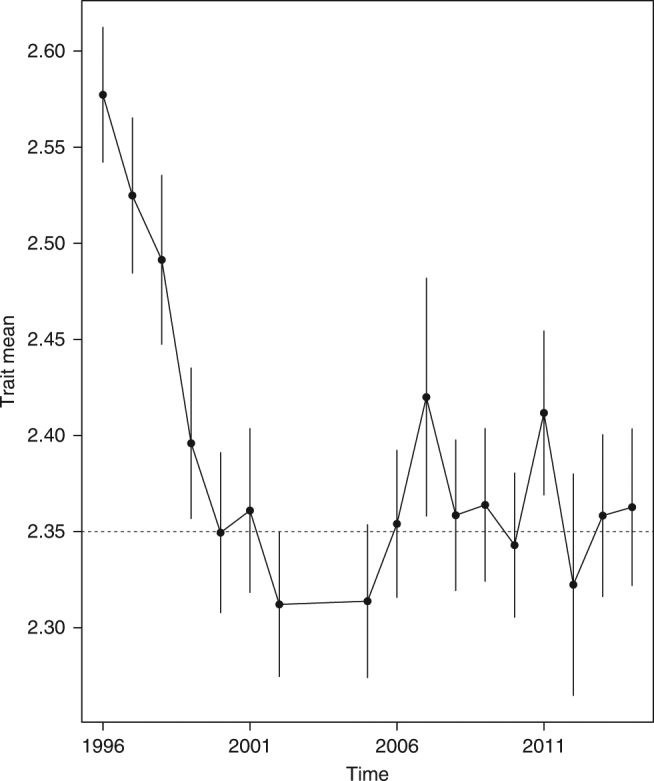
The evolution of log body mass over time, corrected for seasonal variation linked to capture date. We regressed capture date (days away from the center of the breeding season (July 1st)) on log body mass (the dependent variable), having year a factor. Vertical error bars signify one standard error. The data reveal a negative trend in body mass consistent with an OU-model. The dotted line represents the estimated adaptive optimum (*θ*) for log body mass (2.35)

Interestingly, mean annual body mass of juveniles corrected for seasonality also exhibits a negative trend over the study period, and these data also show a much better fit to an OU-model than to a neutral model (Supplementary Table 2, Supplementary Figure 3). Juveniles were born and ringed on site and the observed trend can therefore not be explained by the potential inclusion of migrants. Furthermore, juveniles recaptured as adults were significantly lighter than the ones not recaptured (mean ± SD of those not recaptured: 12.23 ± 2.23 g, mean ± SD of those recaptured: 10.93 ± 1.87 g, linear model: Estimate ± SE = −1.30 ± 0.41, *P* = 0.0016.).

Also, when running a linear regression of body mass throughout the years within each month for adult birds, there is a significantly negative trend within June, July and August (Supplementary Table 3). This demonstrates that body mass decreased significantly from 1996 to 2014, without the possibility for migrants to influence the pattern, as the probability of capturing migrants in June or July is negligible. We acknowledge that without further experimental data, we cannot know the exact mechanisms behind the decrease in body mass, nor why it is apparent both in adults and juveniles.

In order to investigate whether our estimates of survival in the population could have been affected by the inclusion of migrants, we compared birds captured in June/July to birds captured in May/August in terms of proportion recaptured and proportion not recaptured. The proportions were not significantly different from each other (Supplementary Table 4; two-tailed Fisher’s exact test, *P* = 0.18), suggesting that our survival estimates are not merely artifacts of including migrants.

Neto et al.^3^ also seem skeptical towards our conclusion since our results would “constitute an example of exceptionally rapid adaptive evolution in the wild”. Although we agree evolution was fast, we do not consider it to be exceptional: the haldanes calculated from our model are within the normal range of evolutionary rates measured in populations affected by human-induced environmental changes^8^. The half-life we report is as far as we know the shortest estimated from an OU-model, but this is due to the short time interval covered by our data. Also, the estimated selection gradient is within the normal range^9^.

Neto et al.^3^ include a boxplot (Fig. 1 in ref. ^3^) depicting variation in body mass in adult and first year reed warblers in Portugal, showing that body mass is highest in April and from August and onwards. The largest difference between any sample median in Fig. 1 of ref. ^3^ represents a difference of 0.105 natural log units. In comparison, the difference between the initial population in our data and the estimated optimum (from the first ANCOVA model) is 0.23 (0.22 in the original analysis) natural log units, and the difference between the most extreme sample means in our data set is even larger. This suggests that seasonal variation in body mass alone is an insufficient explanation for the decrease in body mass we observe in the Maltese population.

Neto et al.^3^ present the body mass distribution of reed warblers from May to August from their study site in Sweden and point out that the average body mass in the first years of the population in Malta exceeds the mass of the heaviest individual in their Swedish population. It is not clear to us why a single Swedish population should accurately reflect the whole phenotypic range of body mass in this species. The data from Portugal presented by Neto et al.^3^ are indeed containing birds of similar size to what we observe in the Maltese population. Yet, the average body masses of the birds in the initial years were arguably high relative to other populations. We suggest that this may be a result of biased colonization, if the founding population consisted of relatively heavy individuals. Another possibility is that food availability was particularly high in the initial years, and density-dependent effects may be a confounding factor. We acknowledge that we cannot be certain of the origin of the population, and we welcome research to pinpoint the origin of the Maltese population.

Neto et al.^3^ claim the pattern of body size reduction is coincidental and speculates that it is an artifact of the inclusion of migrants in the dataset. We have shown that this is unlikely to be the case. We fail to see how Neto et al.^3^ alternative explanation predicts a trend in body size that is well described by an OU-process. We also note that Neto et al.^3^ do not comment on the fact that the *N*_e_ estimated from the OU model parameters corresponds accurately with our independent molecular estimate of *N*_e_. Our molecular data stems from individuals that were definitely locals; either juveniles or nesting adults captured in June or July. If the data we used in our model selection had been heavily biased by migrants, we would not expect such a close correspondence with the molecular estimate.

We acknowledge, as we did in the original publication, that body mass is a plastic trait. We thank Neto et al.^3^ for pointing out that seasonal variation should be corrected for in analyses of body mass evolution in birds. Doing so puts further strength to our claim that adaptive evolution is likely an important part of the observed trend in body mass in the Maltese reed warbler population.

## Methods

### Statistical analyses

**Fig. 2:**
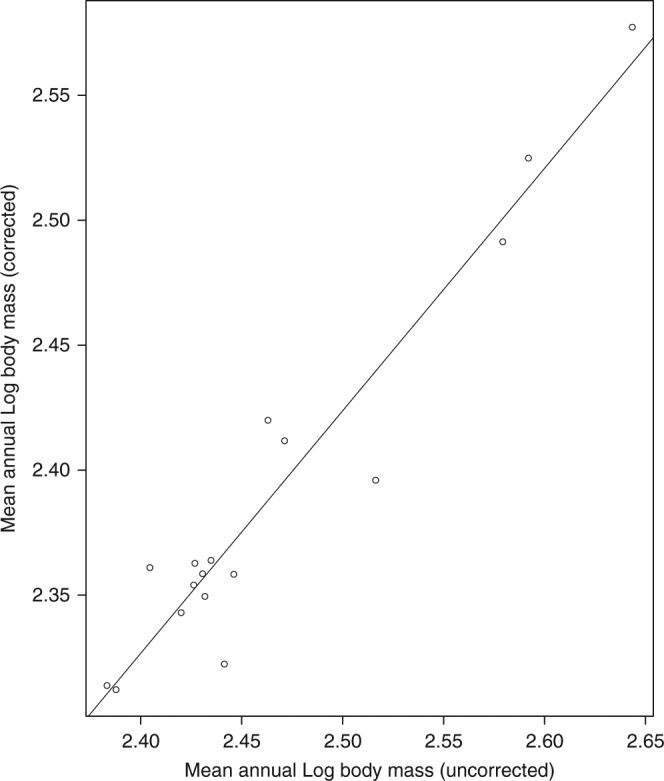
Regression between uncorrected mean annual estimates of Log body mass (from Sætre et al.^1^) and corrected mean annual estimates of log body mass (the values predicted by the first ANCOVA model, where we regressed capture date (days away from the center of the breeding season (July 1st)) on log body mass (the dependent variable), having year a factor). The data show a strong concordance between both estimates (*R*^2^ = 0.92; *P* = 8.29e−10)

We analyzed ANCOVA models where we regressed capture date on log body mass (the dependent variable), having year as a factor. We assumed a common coefficient for how body mass changes as a function of capture date due to the modest sample sizes of measured birds per year. In the first ANCOVA model, capture date was measured as “days away from the center of the breeding season (July 1st)”. We tested the correlation between the annual body mass data used in our original publication and the annual body mass data corrected for seasonal variation with this model, and there is a strong concordance between both estimates (*R*^2^ = 0.92; *P* = 8.29e−10, Fig. 2). In the second ANCOVA model, capture date was implemented as “days after May 1st” as a quadratic term (with the linear term also included in the model). The third model is a linear mixed-effect model implemented using the lme4 package^10^ where log body mass was the dependent variable, year was a fixed effect and month (the month birds where captured) was implemented as a random factor. We note that there are several other non-linear models we could have used, which may have fitted the data better. However, we believe it is best to avoid complex, parameter-rich models given our limited sample size.

For each model, we used the predicted mean and variance to compare the goodness of fit of a neutral (unbiased random walk) and an adaptive (OU) model using the PaleoTS package^11^ in R. We used bias-corrected AICc as a measure of model fit, and to show the relative support for the two models we used Akaike weights (transformations of the AICc scores to make them sum to one). We also conducted a log-likelihood ratio test using the log-likelihood estimates from the models.

### Data availability

The data are available from the Dryad Digital Repository (doi: 10.5061/dryad.kg3hp51).

## Electronic supplementary material

Supplementary Information
