# Supplementary material for: Reply to ‘Inconclusive evidence for rapid adaptive evolution’
Source: Nat Commun. 2018 Jul 10;9:2664. doi: 10.1038/s41467-018-05120-9 (PMC6039529; doi:10.1038/s41467-018-05120-9)
Supplement: Supplementary file 1 — Supplementary Information [file 41467_2018_5120_MOESM1_ESM.pdf]

Supplementary Information

Reply to 'Inconclusive evidence for rapid adaptive evolution'

Sætre *et al.*

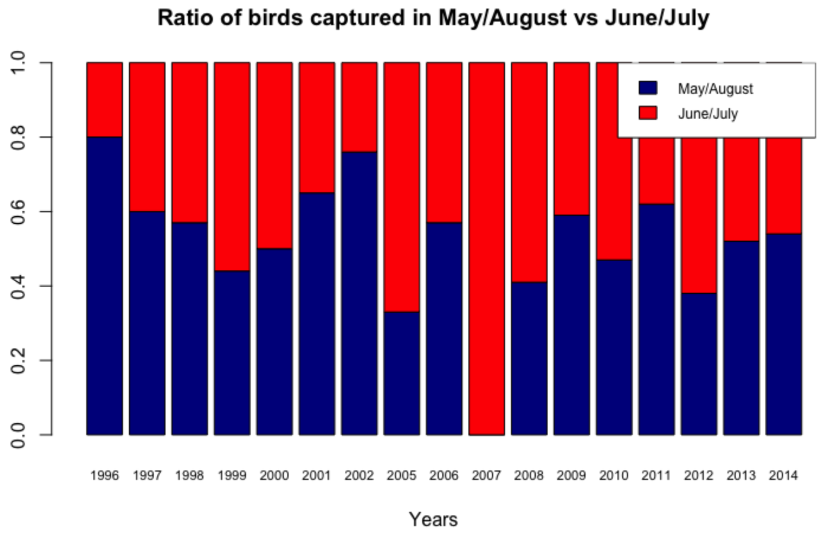

Supplementary Figure 1. Ratio (proportion) of birds captured in May and August to birds captured in June and July in each year.

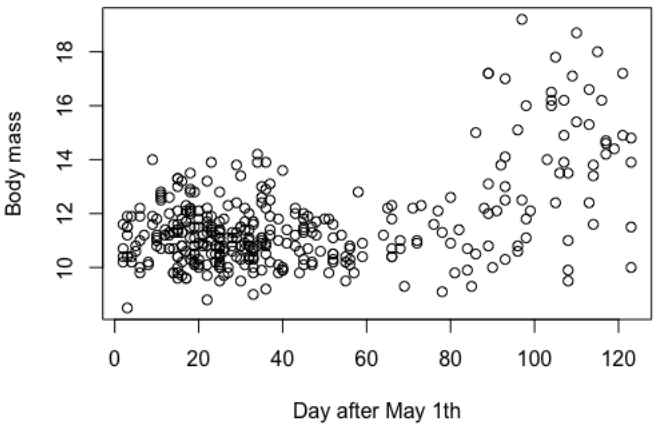

Supplementary Figure 2. Plot showing the relationship between body mass and capture date (day after May 1th). The correlation is highly significant (R^2: 0.23, $P < 2e\text{-}16$).

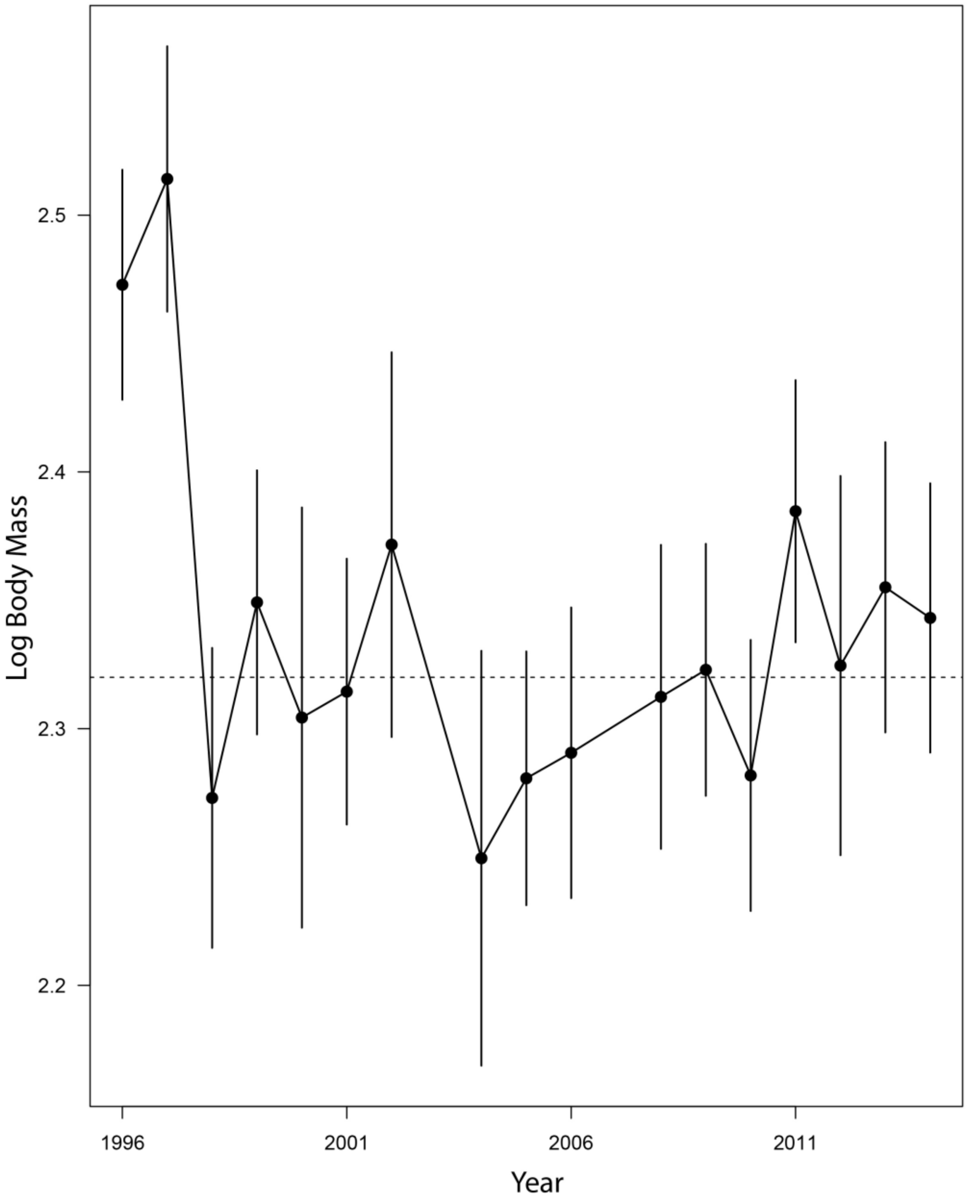

Supplementary Figure 3. The evolution of log body mass over time in juvenile birds, corrected for seasonal variation linked to capture date. We regressed capture date on log body mass (the dependent variable), having year a factor. Capture date was measured as “days away from the center of the breeding season (July 1th)”. Vertical error bars signify one standard error. The data reveal a negative trend in body mass consistent with an OU-model. The dotted line represents the estimated adaptive optimum (θ) for log body mass (2.32).

Supplementary Table 1. Estimates of model fit for a neutral and an adaptive model of evolution for mean body mass corrected for seasonality. We used the estimates and variance of body mass from three different models; the first two are ANCOVA models, where we regressed capture date on log body mass (the dependent variable), having year as a factor. In the first ANCOVA model, the variable capture date was measured as “days away from the center of the breeding season (July 1st)”. In the second ANCOVA model, the variable capture date was measured as "days after May 1st" as a quadratic term. The third model is a linear mixed-effect model where log body mass was the dependent variable, year was the fixed effect and month (month the birds were measured) was implemented as a random factor. For the three models, the adaptive optimum (θ) for log body mass according to the adaptive model is 2.35, 2.43 and 2.44, respectively, the step variance (σ^2_{step}) is 1e-10 for all three models, and the alpha (α), the strength of the restraining force around the optimum, is 0.52, 0.53 and 0.48, respectively. The log-likelihood (logL), number of parameters (K), bias-corrected Akaike Information Criterion (AICc) and Akaike weights suggest that the adaptive model is the more likely model. A likelihood ratio test (LRT), which tests the significance of the improved fit of the adaptive over the neutral model, with the latter treated as the null model, confirmed that the observed changes in body mass are of an adaptive nature. The LRT statistic is distributed as a χ^2, with two degrees of freedom.

Capture date as	Model	logL	K	AICc	Akaike weights	LRT
Continuous term	Neutral	25.32	2	-45.77	0.0001	
	Adaptive	33.06	4	-54.79	0.9999	15.50, *P* = 0.0004
Quadratic term	Neutral	28.01	2	-51.16	0.0068	
	Adaptive	33.73	4	-56.13	0.9932	11.45, *P* = 0.003
Random effect	Neutral	28.23	2	-51.61	0.0497	
	Adaptive	32.95	4	-54.56	0.9503	9.43, *P* = 0.009

Supplementary Table 2. Estimates of model fit for a neutral and an adaptive model of evolution for mean body mass corrected for seasonality in juvenile birds. We regressed capture date on log body mass (the dependent variable), having year a factor. Capture date was measured as "days away from the center of the breeding season (July 1st)". According to the adaptive model, the adaptive optimum (θ) for log body mass is 2.32, the step variance (σ^2_{step}) is 1e-10, and the alpha (α), the strength of the restraining force around the optimum, is 0.58. The log-likelihood (logL), number of parameters (K), bias-corrected Akaike Information Criterion (AICc) and Akaike weights suggest that the adaptive model is the more likely model. A likelihood ratio test (LRT), which tests the significance of the improved fit of the adaptive over the neutral model, with the latter treated as the null model, confirmed that indeed the observed changes in body mass are of an adaptive nature. The LRT statistic is distributed as a χ^2, with two degrees of freedom.

Capture date as	Model	logL	K	AICc	Akaike weights	LRT
Continuous term	Neutral	21.27	2	-37.68	0.033	
	Adaptive	26.19	4	-41.05	0.967	9.84, *P* = 0.007

Supplementary Table 3. Linear regression of body mass to year within each month in our data set. The table shows the model estimates and standard errors (SE), and the corresponding *P*-values.

Month	Estimate ± SE	*P*-value
May	-0.01 ± 0.01	0.440
June	-0.04 ± 0.02	0.018
July	-0.08 ± 0.03	0.021
August	-0.11 ± 0.06	0.049

Supplementary Table 4. Table showing the proportion of birds recaptured (after a minimum of 21 days) and not recaptured, of birds that were first captured in the possible migratory period (May/August), and birds first captured in the center of the breeding season (June/July). The proportions between the two periods are not significantly different from each other (two-tailed Fisher's exact test: *P* = 0.18).

	Not recaptured	Recaptured
Possible migratory period	0.85	0.15
Center of breeding season	0.79	0.21
